# The Effect of Swinging Ball Heads with Different Arrangements in Multi-Point Stretch-Forming Process

**DOI:** 10.3390/ma12030337

**Published:** 2019-01-22

**Authors:** Jian Xing, Yan-yan Cheng, Zhuo Yi

**Affiliations:** 1 Engineering Training Center, Northeast Electric Power University, Jilin 132012, China; xingjian@neepu.edu.cn; 2 Dieless Forming Technology Center, Jilin University, Changchun 130025, China; yizhuo15@mails.jlu.edu.cn; 3 School of Mechanical and Automotive Technology, Jilin Vocational College of Industry and Technology, Jilin 132013, China

**Keywords:** multi-point die, stretch forming, swinging ball head, numerical simulation

## Abstract

To improve the effect of multi-point stretch forming of sheet metal, it is proposed in this paper to replace a fixed ball head with a swinging ball head. According to the multi-point dies with different arrangements, this research establishes finite element models of the following stretch forming, i.e., fixed ball heads with conventional arrangement, swinging ball heads with conventional arrangement, swinging ball heads with declining staggered arrangement, and swinging ball heads with parallel staggered arrangement, and then numerical simulation is performed. The simulation results show that by replacing a fixed ball head with a swinging ball head, the surface indentation of the part formed was effectively suppressed, the stress and tension strain distribution of the part formed was improved, and the forming quality was improved; the thickness of the elastic pad was reduced, the springback was reduced and the forming accuracy was improved; and when the ball head was applied to a multi-point die with staggered arrangement, a better forming result was achieved, where the best forming result was achieved in combining the swinging ball heads with the multi-point die with a parallel staggered arrangement. Forming experiments were carried out, and the experimental results were consistent with the trend of numerical simulation results, which verified the correctness of the numerical simulation.

## 1. Introduction

**Figure 1:**
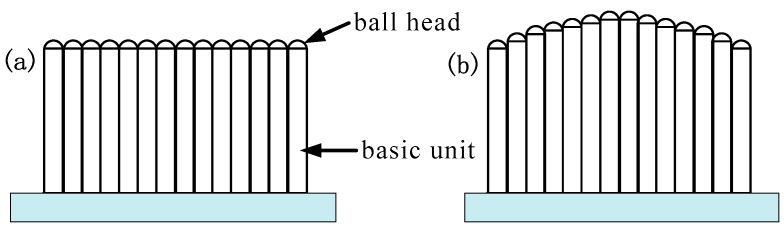
Schematic diagram of multi-point die: (**a**) before adjustment; (**b**) after adjustment.

Multi-point stretch forming (MPSF) [1,2] is a kind of flexible sheet forming method [3] mainly applied to the production of large-size and small-curvature skin parts in the aerospace industry [4]. The main feature of MPSF is the use of multi-point dies instead of solid dies in the process of stretch forming sheet metal. The multi-point die (shown in Figure 1) [5] consists of a series of regularlyarranged and height-adjustable basic units, each of which can be independently digitally adjusted in the vertical direction. The basic units can quickly and flexibly generate the required die profile by a computer control system according to the computer-assisted design (CAD) parameters of the die. The application of only one multi-point die can generate a variety of die profiles, which not only reduces the cost of die manufacturing and improves processing efficiency, but also adjusts the height of the basic unit in real time for die surface compensation [6]. The die profile generated by a multi-point die is actually enveloped by the surface of a plurality of a basic unit’s ball heads and, therefore, the die profile has the feature of discontinuity. Besides, indentation defects are generated on the surface of the formed part during the forming process [7], which affects the surface quality of the formed part [8]. In production, an elastic pad with a certain thickness is usually placed between the multi-point die and the sheet metal according to the material properties, thickness, curvature and other factors to suppress the indentation [9,10]. The thicker the elastic pad, the better the inhibition effect on the indentation [11], but the forming accuracy is reduced [12,13]. Therefore, improving the surface quality and forming accuracy of the formed parts has always been a key issue in the research of the MPSF process. This paper attempts to improve the sheet-forming effect by applying a swinging ball head on the basic units with different arrangements.

## 2. The Forming Principle of a Swinging Ball Head

### 2.1. Geometric Relationship Between Ball Head Radius and Formed Part Surface

The profile of a multi-point die has discontinuity. During the stretch forming process, stress is concentrated at the contact position between sheet metal and ball head to form an indentation, which affects the surface quality of the part. The radius of a basic unit’s ball head directly determines the contact area with the sheet metal and has a great influence on the forming quality

**Figure 2:**
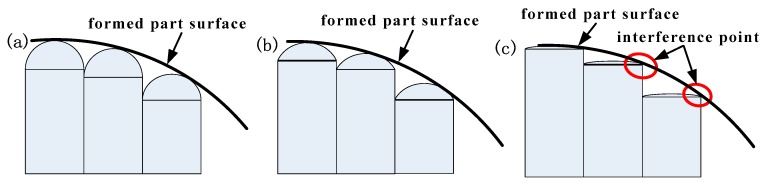
Multi-point dies with different ball head radius: (**a**) small ball head radius; (**b**) large ball head radius; (**c**) ultra-large ball head radius.

Figure 2 is a schematic illustration of the contact between the balls of different radii and the formed part with same curvature. Figure 2a is a die profile formed by a small ball head radius, where the contact area between the formed part surface and the ball head is small, and a large stress is generated at the contact position in the forming process, resulting in a serious local deformation of the formed part surface. Figure 2b is a die profile formed by a large ball head radius. Compared with Figure 2a, the contact area between the formed part surface and the ball head is increased, the load applied by the basic unit is effectively dispersed in forming process, the local deformation caused by the ball head is reduced, and the surface quality of the formed part is increased. As illustrated by Figure 2a,b, the larger the radius of the ball head, the better the surface quality of the formed part. If the radius of the ball head continues to increase as shown in Figure 2c, the formed part surface will not be tangential to the surface of the ball head, and the interference points will be formed in the formed part at the edge of the untangential basic units (the circle in Figure 2c), thereby reducing the surface quality of the formed part surface.

**Figure 3:**
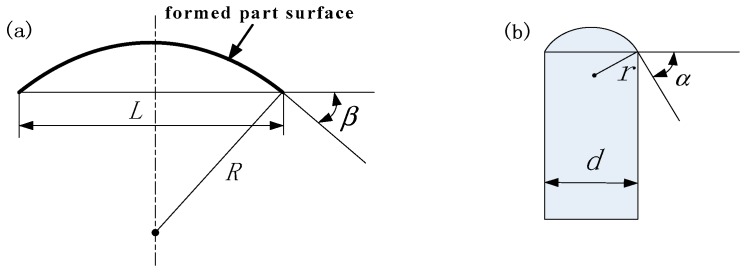
Schematic diagram of formed surface and basic unit’s ball head: (**a**) formed part surface; (**b**) basic unit.

It can be seen from the above analysis that in the process of MPSF, the surface quality of the formed part can be improved only by increasing the radius of the basic unit’s ball head within a certain range. To determine the appropriate radius of the ball head, the geometric relationship between the radius of the ball and the formed part surface is derived by taking a spherical surface as an example, as shown in Figure 3.

In Figure 3a, *L* is the chord length of the formed part surface and *R* is the spherical radius. In Figure 3b, *d* is the length of the basic unit and *r* is the radius of the basic unit’s ball head. If the formed part surface interferes with the surface of the ball head, the interference point firstly appears in the position of the outermost basic unit of the multi-point die (see Figure 2c).Therefore, it is assumed that the basic unit in Figure 3b is located at the outermost side of the multi-point die. If the formed part surface is tangential to the surface of the ball heads of all basic units, according to the geometric relationship, we have:

(1) cosβ≥cosα

(2) $$\cos\beta =\sqrt{1-{\left(\frac{L}{2R}\right)}^{2}}$$

(3) $$\cos\alpha =\sqrt{1-{\left(\frac{d}{2r}\right)}^{2}}$$

By the simultaneous equations of Equations (1)–(3), we have:

(4) $$r\leq \frac{Rd}{L}$$

Equation (4) is the geometric condition that the formed surface is tangent to the ball heads of all basic units of multi-point die. When the geometric condition does not satisfy Equation (4), the sheet metal will interfere with the edge of the basic unit during the forming process.

### 2.2. The Design of the Swinging Ball Head

**Figure 4:**
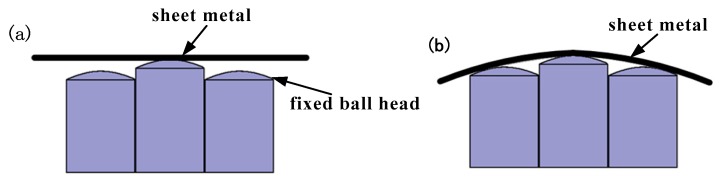

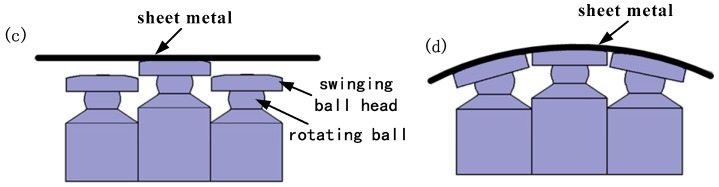
Forming process of fixed ball head and swinging ball head: (**a**) before forming by fixed ball head; (**b**) after forming by fixed ball head; (**c**) before forming by swinging ball head; (**d**) after forming by swinging ball head.

To solve the problem of the interference between sheet metal and the edge of the basic unit caused by the excessively large radius of ball head, it is proposed in this research to design the ball head into a swinging form [14]. The design principle is shown in Figure 4.

It can be seen from Figure 4 that when forming is performed using a fixed ball head, since the radius of the ball head cannot be too large, the contact area between the formed part surface and the ball head is small, and local deformation is easily generated to further form an indentation in the part. When forming is performed using a swinging ball head, under the action of a stretch forming force, the ball head can be swung by the pressing action of the sheet metal, and the swing angle changes according to the curvature of the target part, so that the tangent point between the ball head and the target part can always be at the center of the ball head’s geometry. Since the radius of the swinging ball head can be set to a large value, the contact area between the sheet metal and the ball head is significantly increased, the die profile becomes more continuous, and the load applied by the ball head is effectively dispersed. Therefore, a swinging ball head can effectively improve the surface quality of the formed part.

## 3. The Effect of the Swinging Ball Head on the Forming Result

**Figure 5:**
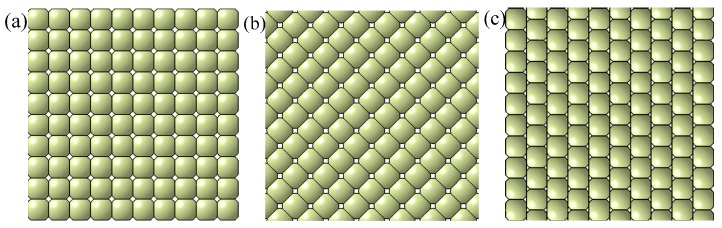
Arrangement modes of basic units: (**a**) conventional arrangement; (**b**) declining staggered arrangement; (**c**) parallel staggered arrangement.

A spherical part was taken as the research object to investigate the effect of swinging ball head on forming result. A fixed ball head and swinging ball head were applied to the multi-point dies (shown in Figure 5) with a conventional arrangement [15], declining staggered arrangement, and parallel staggered arrangement, respectively. The four finite element models were established and numerically simulated, i.e., fixed ball heads with conventional arrangement (FBHC), swinging ball heads with conventional arrangement (SBHC), swinging ball heads with declining staggered arrangement (SBHDS), and swinging ball heads with parallel staggered arrangement (SBHPS). 

The radius of curvature of the spherical part was 1500 mm, the sheet metal was St14 with a size of 1200 mm × 860 mm × 0.7 mm, and the planar size of the forming area was 800 mm × 800 mm. The fixed ball head had a radius of 30 mm and the swinging ball head had a radius of 1500 mm. In order to avoid serious indentation on the surface of the formed part, it was necessary to use an elastic pad on the surface of the multi-point die. Generally speaking, the thicker the elastic pad is, the better the suppressing effect will be [13]. The whole elastic pad with thickness of10mm was used in the multi-point die with fixed ball heads and discrete elastic pads with a thickness of 5 mm were used in the multi-point die with swing ball heads. The stretch forming equipment with 10 clamps on a single side was adopted, with a horizontal force of 7 kN, tilting force of 6.5 kN, vertical force of 10.5 kN. The finite element analysis is completed by software ABAQUS/Explicit. 

St14 was low carbon steel, the isotropic, elastic-plastic constitutive behavior with isotropic hardening was assumed for the sheet metal material. The relevant mechanical properties were set to: density ρ = 7.85 g/cm^3^, elastic modulus *E* = 207 GPa, yield strength σ*_y_* = 176.3 MPa, Poisson’s coefficient ν = 0.28, strain-hardening coefficient *n* = 0.247, and strength coefficient *K* = 596 MPa. The sheet metal was modeled with shell element S4R. Polyurethane with a Shore A hardness of 85 was chosen as theelastic pad material. Polyurethane was an elastomer, which behaved in a non-linear elastic manner and, generally, was assumed to be incompressible. The elastic pad was described by the Mooney–Rivlin hyper-elastic material model in our numerical simulations. The nominal stress–strain relationship of polyurethane was obtained by a uniaxial compression test. The elastic pad was modeled using C3D8R solid elements. The ball heads were modeled using R3D4 elements. A general contact algorithm was applied in all the numerical analysis. Coulomb’s model was followed in the problems of the frictions. The friction coefficients of elastic pad-to-sheet metal and elastic pad-to-multi-point die were set as 0.57, which was tested through a friction experiment. Considering the symmetry of the model and the computational time, only a quarter of the finite element model was built. The sheet metal was constrained with symmetrical boundary conditions and the die was fixed in the simulation process. Finite element mesh density of sheet metal was 1 mm.

### 3.1. Surface Quality Analysis

**Figure 6:**
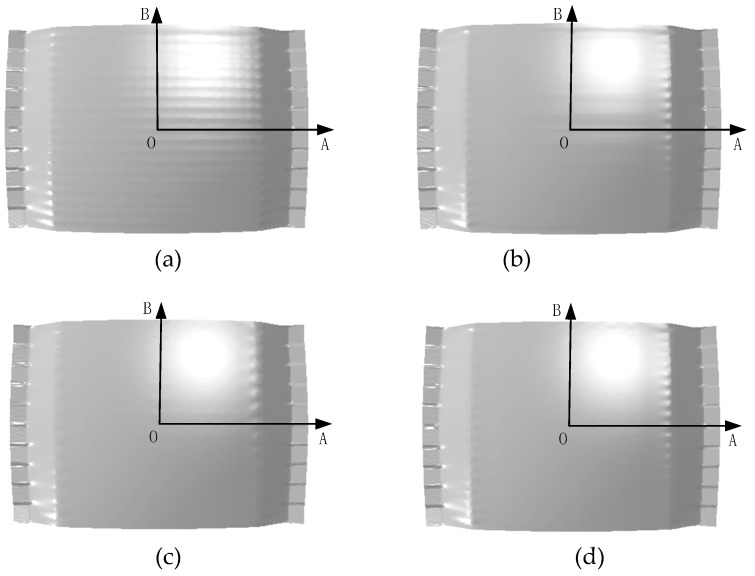
Light maps of the formed parts: (**a**) formed by fixed ball heads with conventional arrangement (FBHC); (**b**) formed by swinging ball heads with conventional arrangement (SBHC); (**c**) formed by swinging ball heads with declining staggered arrangement (SBHDS); (**d**) formed by swinging ball heads with parallel staggered arrangement (SBHPS).

Figure 6 is the light map of the simulation formed parts. Figure 6a corresponds to the FBHC for the 10 mm-thick elastic pad. As shown in Figure 6a, due to the small radius of the fixed ball head, the stress is concentrated at the contact point, the indentation is relatively obvious, and there is obvious stretch groove along the stretch direction. Figure 6b is the formed part corresponding to the SBHC. When using a 5 mm-thick elastic pad, the surface forming quality is remarkably improved without significant indentation, but there is a slight stretch groove along the stretch direction in the middle of the formed part. Figure 6c,d show the formed parts corresponding to the SBHDS and the SBHPS, respectively, for a5 mm-thick elastic pad. The surface quality of the formed parts in Figure 6c,d have been improved compared with those in Figure 6a,b, and there is no obvious indentation and stretch groove. Through a detailed comparison between Figure 6c,d, it can be seen that the surface of the formed part in Figure 6c is slightly rough, and its smoothness is worse than that in Figure 6d, so the formed part in Figure 6d has the best surface quality. There are two reasons for the gradual improvement of the surface quality of the formed parts from Figure 6a to Figure 6d. Firstly, the use of swinging ball head effectively increases the contact area between the sheet metal and the ball head, so that the die profile has a higher continuity; secondly, the use of staggered dies suppresses the forming defects.

### 3.2. Stress Analysis

**Figure 7:**
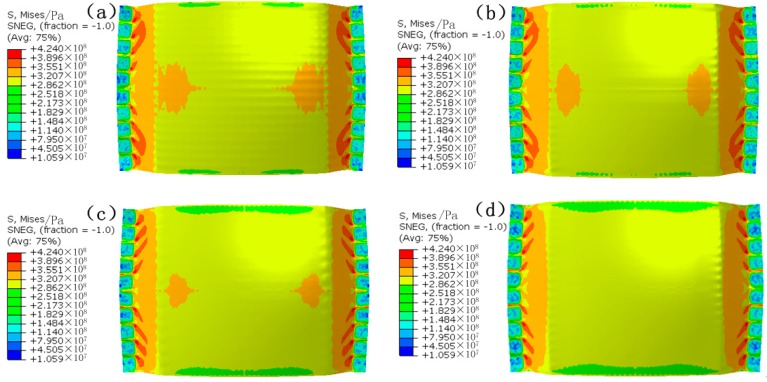
Stress distribution of the formed parts: (**a**) formed by FBHC; (**b**) formed by SBHC; (**c**) formed by SBHDS; (**d**) formed by SBHPS.

Figure 7 is the stress distribution. It can be seen that the uniformity of the stress distribution of the FBHC is the worst, and that of the SBHPS is the best. A stress concentration area occurs in the direction in which the formed part is stretched near the edge of the die. This is due to the fact that the vertical force causes the sheet metal to interact with the edge of the die, but the stress concentration area gradually decreases as the die arrangement changes, and eventually disappears.

**Figure 8:**
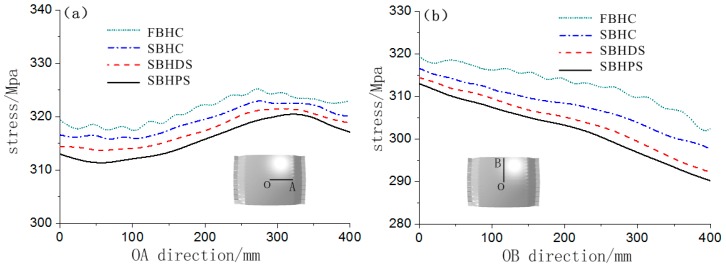
Stress distribution curves of the formed parts: (**a**) along the OA direction; (**b**) along the OB direction.

In order to quantify and compare the stress, the stress value of the symmetry axis of the formed part was extracted, as shown in Figure 8. It can be seen that the stress value increases first along the OA direction, and decreases near the edge position, which is related to the occurrence of the stress concentration area in Figure 7. Along the OB direction, the stress value gradually decreases. At the same position on the symmetry axis, the stress value corresponding to the FBHC is the largest, and the stress value corresponding to the SBHPS is the smallest. A relatively large fluctuation is observed in the stress curve corresponding to the fixed ball heads, indicating that the stress distribution is not uniform, and this phenomenon is caused by the large local deformation of the corresponding position of the ball head. The stress curve of the swinging ball heads is smooth, indicating that the stress distribution is uniform. The maximum stress of the FBHC and the SBHPS along the OA direction are 326.0 MPa and 320.7 MPa, respectively, and those along the OB direction are 319.3 MPa and 313.0 MPa, respectively.

### 3.3. Tension Strain Analysis

**Figure 9:**
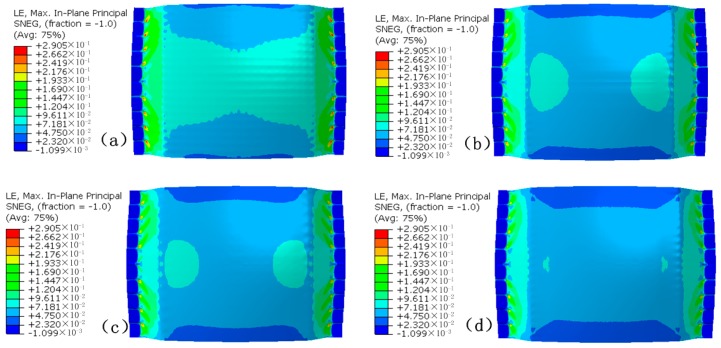
Tension strain distribution of the formed parts: (**a**) formed by FBHC, (**b**) formed by SBHC, (**c**) formed by SBHDS and (**d**) formed by SBHPS.

Figure 9 shows the tension strain distribution of the formed parts. It can be seen from Figure 9 that the tension strain distribution corresponding to the FBHC has the worst uniformity, and that corresponding to the SBHPS has the best uniformity. The tension strain distribution trend in Figure 9 is consistent with the stress distribution trend in Figure 7. 

**Figure 10:**
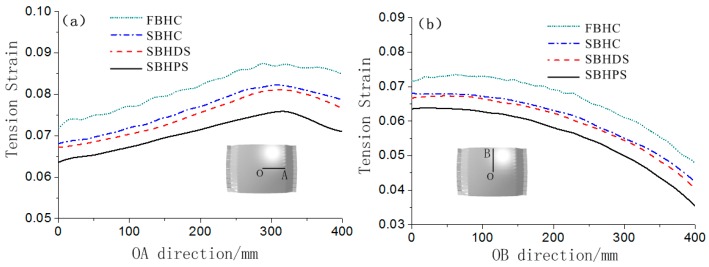
Tension strain distribution curves of the formed parts: (**a**) along OA direction; (**b**) along OB direction.

The tension strain values of the symmetry axis of the formed parts are extracted for quantitative comparison, as shown in Figure 10. It can be seen that along the OA direction, the tension strain value increases at first and then decreases, which is consistent with the stress distribution in Figure 8; along the OB direction, the tension strain value gradually decreases. At the same position of the symmetry axis, the tension strain value corresponding to the FBHC is the largest, and that corresponding to the SBHPS is the smallest. A relatively large fluctuation is observed in the tension strain curve corresponding to the fixed ball heads, indicating that the tension strain distribution is not uniform. The tension strain curve of the swinging ball heads is smooth, indicating that the tension strain distribution is uniform. The maximum tension strains of the FBHC and the SBHPS along the OA direction are 0.088 and 0.076, respectively, and those along the OB direction are 0.074 and 0.064, respectively.

### 3.4. Springback Analysis

**Figure 11:**
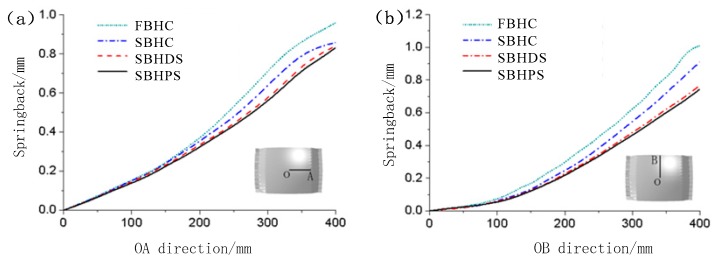
Springback distribution curves of the formed parts: (**a**) along OA direction; (**b**) along OB direction.

Figure 11 shows the springback distribution curves on the symmetry axis of the formed parts. It can be seen that along the OA and OB directions, at any same position of the symmetry axis, the springback of the FBHC is larger than that of other arrangements of multi-point dies; and that very close springbacks are observed in the formed parts corresponding to the SBHDS and the SBHPS, but the springback of the latter is slightly smaller. There are two reasons for the above phenomenon. Firstly, the multi-point die with fixed ball heads used a 10 mm-thick elastic pad, while the multi-point die with swinging ball heads used a 5 mm-thick elastic pad, so that the reduction of the thickness of the elastic pad reduces the springback of the formed part. Secondly, the change in the arrangement of the multi-point die also reduces the springback of the formed part.

## 4. Forming Experiments

**Figure 12:**
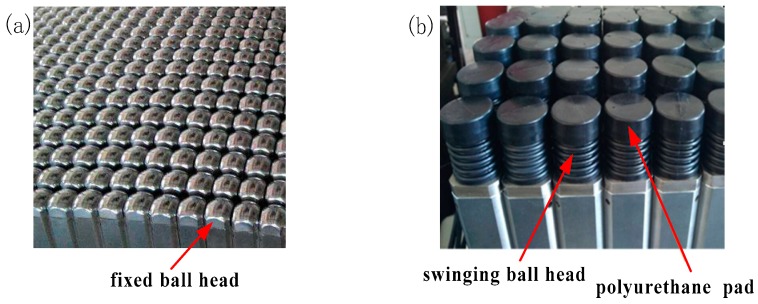
Multi-point dies with fixed ball heads and swinging ball head: (**a**) fixed ball head; (**b**) swinging ball head.

To verify the forming effect of the multi-point die with fixed ball heads and swinging ball heads, stretch forming experiments were performed. Limited by the experimental conditions, only the multi-point die with conventional arrangement was conducted, as shown in Figure 12. Figure 12a shows the multi-point die of FBHC, where the radius of the ball head was 30 mm and a 10 mm-thick elastic pad was used to avoid excessive indentation during forming. Figure 12b is the multi-point die of SBHC, where the radius of the ball head was 1500 mm, the diameter of the cylindrical rotating unit below the ball head was 38 mm, and a 5 mm-thick polyurethane elastic pad was used on the surface of the swinging ball head. The target part had a radius of 1500 mm, the planar size of the multi-point die was 1200 mm× 1600 mm, and the sheet metal had a size of 1200 mm× 2000 mm× 0.7 mm.

**Figure 13:**
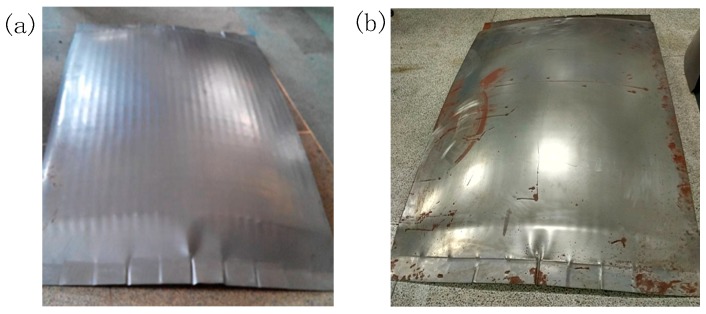
Experimental parts for stretch forming: (**a**) formed by FBHC; (**b**) formed by SBHC.

Figure 13 shows the picture of the experimental parts. Figure 13a is the formed part corresponding to the FBHC, where the surface indentation is obvious, and a distinct stretch groove is formed along the stretch direction. Figure 13b shows the formed part corresponding to the SBHC, where the smoothness of the surface is improved, the indentation is very slight, and there is no obvious stretch groove. The forming results are consistent with the trend of numerical simulation results, indicating that the multi-point die with swinging ball heads can effectively suppress the occurrence of indentation on the surface of the formed part.

## 5. Conclusions

- The replacement of a fixed ball head with a swinging ball head can form a more continuous die profile, effectively suppressing the surface indentation of the formed part and improving the forming quality.
- The use of a swinging ball head can reduce the use of an elastic pad, reduce the springback, and improve the forming accuracy.
- A better forming effect can be achieved by applying a swinging ball head to the multi-point die with a staggered arrangement, where the best forming effect can be achieved in using the multi-point die with SBHPS.
- The experimental results are consistent with the trend of numerical simulation results, verifying the correctness of the numerical simulation results and providing a valuable reference for the development of new multi-point dies.
